# Corneal Refractive Surgery in Patients with History of Optic Neuritis

**DOI:** 10.18502/jovr.v14i4.5445

**Published:** 2019-10-24

**Authors:** Majid Moshirfar, William D. Wagner, Steven H. Linn, Tanner W. Brown, Jackson L. Goldberg, Aaron T. Gomez, Yasmyne C. Ronquillo, Phillip C. Hoopes

**Affiliations:** ^1^John A. Moran Eye Center, Department of Ophthalmology and Visual Sciences, University of Utah School of Medicine, Salt Lake City, UT, USA; ^2^HDR Research Center, Hoopes Vision, Draper, UT, USA; ^3^Virginia Commonwealth University School of Medicine, Richmond, VA, USA; ^4^The University of Texas Health Science Center at Houston School of Medicine, Houston, TX, USA; ^5^The University of Texas Rio Grande Valley School of Medicine, Edinburg, TX, USA

**Keywords:** Laser vision surgery, LASIK, Multiple sclerosis, Optic Neuritis, PRK

## Abstract

**Purpose:**

The purpose of this study was to evaluate the risk of recurrence of optic neuritis after corneal refractive surgery in patients with a history of optic neuritis and to examine the safety and efficacy of the procedure in this population.

**Methods:**

This was a retrospective chart review of patients with a history of optic neuritis who underwent laser-assisted in situ keratomileusis (LASIK) or photorefractive keratectomy (PRK) at a single tertiary center from June 1996 to December 2014. Fifteen eyes of 14 patients were included in this study. Visual acuity before and after the surgery was recorded. Patients were followed-up for over five years postoperatively for the recurrence of optic neuritis.

**Results:**

The average LogMAR best corrected visual acuity (BCVA) preoperatively was 0.12 ± 0.19 (–0.10 to 0.60) and postoperatively was 0.06 ± 0.10 (–0.10 to 0.30). No eyes lost lines of BCVA. The average LogMAR uncorrected distance visual acuity (UDVA) after surgery was 0.12 ± 0.13 (0.00 to 0.48). Twenty-eight percent of patients reached a UDVA of 20/20 or better after refractive surgery. Optic neuritis recurred in 3/15 (20%) eyes and 3/14 patients (21%).

**Conclusion:**

While corneal refractive procedures appear safe in patients with a history of optic neuritis, our data suggest that their efficacy may be reduced.

##  INTRODUCTION

There are various potential causes of optic neuritis, including multiple sclerosis (MS), systemic lupus erythematosus, antiphospholipid syndrome, Sjogren's syndrome, and multiple vasculitides.^[1,2,3,4]^

Optic neuritis is most commonly associated with MS, with approximately 50% of patients experiencing an episode during their lifetime.^[5]^ The most common pathologic basis for optic neuritis is immune-mediated inflammatory demyelination of the optic nerve.^[6]^ Previous research has demonstrated disturbances during corneal wound healing in various growth factors, cytokines, and inflammatory cells commonly involved in the pathophysiology of optic neuritis.^[7]^ While various studies have evaluated outcomes of refractive procedures in patients with other autoimmune diseases, there is limited data on patients with a previous history of optic neuritis.^[8,9]^ The aim of this study was to determine the risk of recurrence in patients with a previous history of optic neuritis after corneal refractive surgery. Additionally, we evaluated the safety and efficacy of corneal refractive surgery in this population.

##  METHODS

This was a retrospective chart review of all patients with a history of optic neuritis, who underwent laser-assisted in situ keratomileusis (LASIK) or photorefractive keratectomy (PRK) at a single tertiary center from June 1996 to December 2013. A single surgeon performed all the procedures. At the time of surgery, the patients had experienced their most recent episode of optic neuritis at least two years previously.

Patients included in this study underwent complete refractive surgery workup to rule out herpes, keratoconus, posterior or anterior segment pathology, pars planitis, previous uveitis, or corneal ectasia. Preoperative review of systems was negative for any MS-related symptoms, including bladder dysfunction, motor or sensory disturbances, or visual involvement.

Each patient received approval for LASIK or PRK through consultation with a neurologist and/or family physician who determined that there was no active disease at the time of surgery. All patients underwent standard preoperative refractive workup including uncorrected distance visual acuity (UDVA), best corrected distance visual acuity (CDVA), manifest and cycloplegic refractions, applanation tonometry, slit lamp

biomicroscopy, indirect ophthalmoscopy, pachymetry, and corneal topography. Risks and benefits of the procedure were explained to the patients, and all patients provided written informed consent.

Twelve of the thirteen LASIK procedures were performed using the VISX excimer laser (Santa Clara, California, USA) with ablation zones of 6–8 mm and flap creation using the Hansatome microkeratome (Bausch & Lomb, Rochester, New York, USA). One procedure was performed using a 400 Hz excimer laser (Alcon, Fort Worth, Texas, USA) with flap creation performed using Alcon's FS200 femtosecond laser. There were no intraoperative complications. Patients were routinely examined one day, one week, and one, three, six, twelve, and twenty-four months after the surgery. Postoperatively, patients received the standard pharmacological regimen, which included one week of topical prednisolone acetate 1% four times a day and a third- or fourth-generation fluoroquinolone four times a day, as well as frequent doses of preservative-free topical lubricants for several months.

The PRK procedure consisted of scoring the corneal epithelium using an 8.0-mm trephine with alcohol-assisted epithelial debridement. Laser ablation with a 6.0 mm optical zone and an 8-mm blend was performed using a VISX excimer laser. Patients then received a bandage contact lens with fluoroquinolone and prednisolone acetate 1% drops, each four times a day. After the first week, the bandage contact lens and antibiotic drops were discontinued, and prednisolone acetate 1% was continued four times a day for three additional weeks. Following this, the steroid was changed to fluorometholone 0.1%, whose dose was tapered over the course of additional eight weeks. Postoperative follow-up was performed after one day, one week, and one, three, and six months.

Patients were followed over five years postoperatively and data collected included time from LASIK or PRK to the diagnosis of optic neuritis and visual acuity. The recurrence of optic neuritis was diagnosed clinically and confirmed by direct visualization of a pale optic nerve. In patients who developed recurrence postoperatively, the UDVA and CDVA before and after the episode were recorded. Visual acuity was converted to LogMAR as described by Holladay.^[10]^

##  RESULTS

A total of 14 patients (15 eyes) underwent surgery. PRK was performed on 4 eyes and LASIK was performed on 11 eyes. Of the 15 eyes, 6 were right and 9 were left. The ratio of female to male patients was 11:3. The age of the patients ranged from 23 to 55 years, with a mean age of 34 years. Nine of the fourteen patients had been previously diagnosed with MS. The cause of optic neuritis was not known in the remaining five patients. All eyes demonstrated optic nerve pallor on preoperative funduscopic examination, except one patient in whom no optic nerve pallor was seen. Refractive errors corrected included one hyperopic eye, two eyes with significant astigmatic error, and 12 myopic eyes ranging from –2.50 D to –10.00 D [Table 1].

**Table 1:** Patients' information.


**Patient**	**Eye**	**Age**	**Sex**	**Nerve Pallor**	**MS**	**Preop BCVA**	**Surgery**	**Postop UDVA**	**Postop BCVA**	**Recurrence**	**Time to Recurrence**	**BCVA Post ON Recovery**
1	OD	34	Female	Y	Y	20/20	LASIK	20/20	20/20		
	OS		Y	Y	20/20	LASIK	20/20	20/20		
2	OD	27	Female	Y	N	20/30	LASIK	20/30	20/25		
3	OD	41	Female	Y	Y	20/25	PRK	20/25	20/25	Yes	3 years	20/40
4	OS	34	Male	Y	N	20/50	LASIK	20/30	20/30		
5	OD	39	Male	Y	Y	20/30	LASIK	20/30	20/30	Yes	4 years	20/60
6	OS	33	Female	Y	Y	20/20	LASIK	20/20	20/20		
7	OD	29	Female	Y	Y	20/25	LASIK	20/25	20/25		
8	OS	29	Female	Y	Y	20/80	LASIK	20/60	20/40		
9	OS	23	Female	Y	N	20/40	LASIK	20/30	20/25		
10	OS	33	Male	Y	Y	20/20	PRK	20/20	20/20		
11	OS	55	Female	N	N	20/15	PRK	20/80	20/15		
12	OS	33	Female	Y	N	20/20	LASIK	20/25	20/20		
13	OS	33	Female	Y	Y	20/20	PRK	20/25	20/20		
14	OD	35	Female	Y	Y	20/25	LASIK	20/25	20/20	Yes	5 years	20/40
	
	
MS, multiple sclerosis; BCVA, best corrected visual acuity; LASIK, laser-assisted in situ keratomileusis; N, no; OD, right eye; OS, left eye; PRK, photorefractive keratectomy; UDVA, uncorrected distance visual acuity; Y, yes; Postop, postoperative												

**Figure 1:**
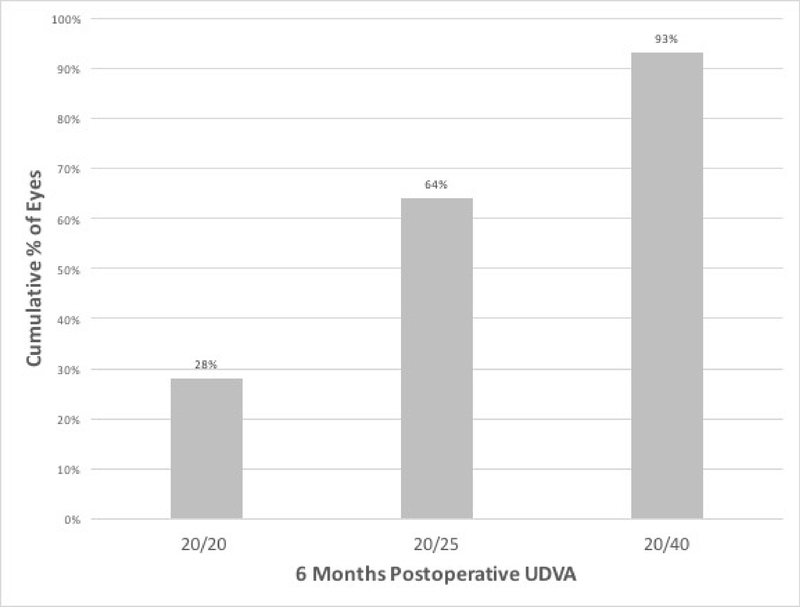
Postoperative uncorrected distance visual acuity (UDVA). Cumulative UDVA at six months postoperatively.

**Figure 2:**
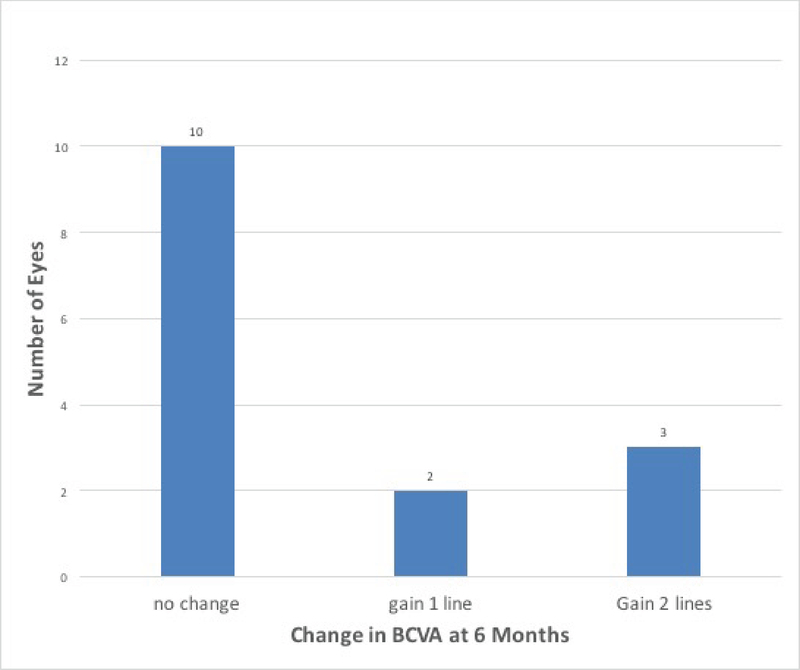
Change in best corrected visual acuity (BCVA). Change in BCVA preoperatively versus six months postoperatively.

Postoperative refraction revealed 10 patients (66.6%) with emmetropia and 4 (26.6%) with residual refractive error (+1.00 D to –2.13 D SEQ). Visual acuity was measured in all patients within six months postoperatively. The average LogMAR UDVA after surgery was 0.12 ± 0.13 (0.00 to 0.48). Twenty-eight percent of patients reached a UDVA of 20/20 or better after refractive surgery, excluding the eye that was targeted for monovision from the calculation [Figure 1]). The UDVA in all patients was 20/40 or better, except in one patient with monovision and in another with residual refractive error. Both the patients improved to 20/40 or better with correction. The average LogMAR BCVA preoperatively was 0.12 ± 0.19 (—0.10 to 0.60) and postoperatively was 0.06 ± 0.10 (–0.10 to 0.30). No eye lost lines of CDVA. Ten patients had no change in CDVA, two patients gained one line, and three patients gained two lines [Figure 2].

Three of the 15 eyes (20%) experienced recurrence of optic neuritis postoperatively. The recurrence occurred in one patient three years after PRK and two patients after LASIK at the fourth and fifth years, respectively. Each of these three patients experienced persistent vision loss. CDVA measured after resolution of acute optic neuritis was 20/40 in two patients and 20/60 in the third patient.

##  DISCUSSION

While various studies have examined the increased risk of developing optic neuropathy after intraocular surgery, there is limited literature on the risk of developing optic neuritis.^[11,12]^ Our study examined patients with a history of optic neuritis in order to determine recurrence rates, visual outcomes, and safety of refractive surgery.

It is well established that optic neuritis typically affects relatively young women. In this study, 11/14 patients (78.5%) were women, compared to 77.2% reported in the optic neuritis treatment trial (ONTT).^[5]^ In a retrospective chart review performed at the Mayo Clinic, the mean age of patients with optic neuritis was 31 years, which is marginally lower than the mean age of our sample population (34 years).^[13]^ Of note, only two patients were over 40 years of age. Based on these demographics, our study population appears to serve as an appropriate sample of the general population of patients with optic neuritis.

Interestingly, one patient in our sample had a history of bilateral optic neuritis which is described as being less prevalent than unilateral optic neuritis.^[14,15]^ In the ONTT, out of the patients who initially presented with unilateral optic neuritis, 9% subsequently developed bilateral symptoms. Bilateral optic neuritis appears to have a good prognosis with recovery of vision if treated appropriately.^[15]^ Notably, our patient with bilateral optic neuritis did not have recurrence and improved to a CDVA of 20/20 in both eyes after surgery.

Although, to the best of our knowledge, no studies have evaluated visual outcomes after refractive surgery in patients with a history of optic neuritis, Hashemi et al investigated visual outcomes in 15 eyes of eight patients with MS after LASIK.^[16]^ Our results are comparable with that reported in the aforementioned study, as average postoperative UDVA and CDVA were approximately 20/25 when excluding the single eye that was targeted for monovision based on calculation of UDVA. While the percentage of patients in the general population with UDVA of 20/20 or better after LASIK or PRK has been reported as 42– 48%, only 28% of patients in our study reached this outcome.^[17]^ Thus, our study is consistent with previous reports and suggests the safety of refractive procedures in patients with a history of optic neuritis, but may indicate decreased efficacy.

Previous studies have reported an occurrence of optic neuritis shortly after refractive surgery; therefore, one of the aims of this study was to evaluate the risk of recurrence of optic neuritis after these procedures.^[12]^ Of note, the ONTT offered long-term follow-up of patients after an episode of optic neuritis. After five years of follow-up, 30% of patients had experienced recurrence of optic neuritis, while our results showed recurrence after refractive surgery in 30% of eyes.^[18]^ Our data does not show an increased risk of recurrence of optic neuritis after refractive surgery compared to the general population; however, subsequent studies with larger sample sizes and inclusion of matched controls are needed to adequately address this comparison.

While most patients in our study had a history of MS, five patients reported no history of conditions associated with optic neuritis. The ONTT found that patients who met the clinical criteria for optic neuritis carried a 50% risk of developing MS over a 15-year follow-up period.^[19]^ It remains unclear whether optic neuritis in the five patients in our study was due to a demyelinating process or another underlying disease yet to be identified. Further studies are needed to evaluate the outcomes of refractive surgery and the risk of recurrence in patients with non-demyelinating systemic conditions related to optic neuritis.

In populations associated with increased risk of recurrence of optic neuritis, it is important to consider measures of vision other than standard visual acuity. Contrast sensitivity can be used to detect deficits in vision that would otherwise be missed using a standard Snellen chart.^[20]^ Optic neuritis is a known risk factor for loss of contrast sensitivity, with up to 78% of patients experiencing persistent impairment even after resolution of active disease, regardless of recovery of visual acuity.^[18]^ Additionally, refractive surgery has been shown to negatively impact contrast sensitivity.^[21,22]^ The presence of both risk factors and the possible compounding effects on visual outcomes warrants further investigation.

Absolute and relative contraindications to refractive surgery have been suggested to optimize patient outcomes and reduce postoperative complications. Although active, uncontrolled autoimmune diseases are considered absolute contraindications, studies have shown that patients with a variety of well-controlled diseases can safely and effectively undergo surgery.^[8]^ Importantly, patients in this study were only included if they met specific criteria indicating control of disease. In order to achieve acceptable outcomes in patients with a history of optic neuritis who wish to undergo refractive surgery, clinicians must ensure appropriate preoperative evaluation of patients. All patients should receive surgical clearance by a neurologist or physician with experience in the diagnosis and management of optic neuritis. Additionally, a comprehensive informed consent should include a discussion about the paucity of data available regarding the impact of optic neuritis on efficacy of surgery, recurrence of disease, and possible loss of contrast sensitivity.

##  Financial Support and Sponsorship

None of the authors have financial interest in any products or methodology mentioned in this manuscript. This research has been supported by Research to Prevent Blindness (New York, New York).

##  Conflicts of Interest

There are no conflicts of interest.
